# Enhancement of Muscle Shortening Torque Preloaded with Muscle Lengthening is Joint-Specific

**DOI:** 10.5114/jhk/161729

**Published:** 2023-04-20

**Authors:** Marzouq K. Almutairi, Gary R. Hunter, Donald H. Lein, SoJung Kim, David R. Bryan, Mario Inacio, Christopher P. Hurt, William Reed, Harshvardhan Singh

**Affiliations:** 1 Department of Physical Therapy, University of Alabama at Birmingham, Birmingham, AL, USA.; 2 Department of Physical Therapy, Qassim University, Qassim, Saudi Arabia.; 3 Department of Nutrition Sciences, University of Alabama at Birmingham, Birmingham, AL, USA.; 4 Department of Kinesiology, Rowan University, Glassboro, NJ, USA.; 5 Department of Physical Education and Sports Science, University of Maia-ISMAI, Maia, Portugal.; 6 Faculty of Health and Life Sciences, Oxford Brookes University, Oxford, UK.

**Keywords:** plyometric, potentiation, stretch shortening cycle, muscle performance

## Abstract

Our cross-sectional study aimed to investigate joint specificity of concentric muscle torque enhancement after a maximum eccentric contraction for the knee versus ankle joints across two different movement velocities (120°/s and 180°/s). After a familiarization session, 22 healthy young adults randomly performed concentric (CONC) and maximum eccentric preloaded concentric (EccCONC) muscle strength tests of the knee extensors and ankle plantar flexors of the non-dominant leg on an isokinetic strength testing device. We calculated the ratio between EccCONC and CONC (EccCONC/CONC) for all the conditions as the marker of concentric muscle torque enhancement. Separate two-way (joints x velocity) within repeated measures ANOVAs were used to determine joint-specific torque differences at 120°/s and 180°/s. CONC and EccCONC were greater for the knee extensors versus ankle plantar flexors at 120°/s and 180°/s (32.86%–102%; p < 0.001 for both); however, EccCONC/CONC was greater for the ankle plantar flexors than knee extensors at 120°/s (52.4%; p < 0.001) and 180°/s (41.9%; p < 0.001). There was a trend of greater EccCONC/CONC for the knee extensors at 180°/s than 120°/s (6.6%; p = 0.07). Our results show that greater concentric muscle torque enhancement after a maximal eccentric contraction occurs for the ankle plantar flexors versus knee extensors. Whether the joint- specificity of concentric muscle torque enhancement after a maximal eccentric contraction differentially affects sports performance is unknown. Our data provide a reference framework to investigate joint-specific concentric muscle torque enhancement for general and clinical athletic populations.

## Introduction

A stretch-shortening cycle i.e., enhancement of muscle shortening/concentric torque after muscle lengthening/eccentric contraction (Cavagna et al., 1968; Seiberl et al., 2015) is considered one of the several basic muscle actions which play a vital role in human locomotion (Nicol et al., 2006) and sports performance (McGuigan et al., 2006). The exact mechanism of enhanced concentric muscle torque after eccentric contractions is still unclear (Seiberl et al., 2015; Tomalka et al., 2020); however, it is well-established that the muscle-tendon complex stores energy during the muscle eccentric phase of walking/running/jumping to use it in enhanced force output during its eventual concentric phase (Cavagna et al., 1968; Edwen et al., 2014; Hunter et al., 2015). In addition to elastic energy storage and use, muscle activation during the eccentric phase allows adequate time for the muscle to reach a greater level of activation before its concentric phase begins (Bobbert and Casius, 2005). Thus, the muscle contractile properties during its eccentric phase can enhance the torque output of its concentric contractions (Turner and Jeffreys, 2010).

Muscle contractile tissues respond differentially to muscle loading during the eccentric phase (Fukunaga et al., 2001), suggesting that enhancement of concentric muscle torque may vary between muscles. In addition, factors such as muscle strength and the amount of the eccentric load can differentially affect the enhancement of concentric muscle torque in a joint-specific way (Nicol et al., 2006). Various other physiological factors such as respective muscle-tendon length and the muscle fiber type can also differentially affect the generation of joint-specific enhancement of concentric muscle torque (Hunter et al., 2015). It is postulated that an interplay of all of these factors could be affected by joint-specificity; however, little is known regarding lower extremity joint-specific comparisons of enhanced concentric muscle torque.

Knowledge of joint-specific differences for the enhanced concentric muscle torque is vital because it may help inform joint-specific contributions to sports performance and various functional tasks. For example, a greater degree of enhancement of concentric muscle torque at the ankle could positively affect propulsive force production during activities such as jumping (Farris et al., 2016), walking or running (Edwen et al., 2014) which, in turn, may affect sports performance or participation in free-living physical activity (Hunter et al., 2021); while the degree of enhanced concentric muscle torque at the knee could affect rapid balance response following a moderate to severe balance perturbation (Cheng, 2016). Thus, an understanding of joint-specific differences of enhanced concentric muscle torque, especially for joints of the lower limb, may provide insights into understanding sports or physical activity performance and planning for joint-specific rehabilitation protocols.

Movement velocity is one of the critical factors that affect the enhanced concentric muscle torque (Blanpied et al., 1995; Svantesson and Grimby, 1995; Svantesson et al., 1994). In fact, previous studies have used 120°/s and 180°/s to examine enhancement of concentric muscle torque after eccentric loading (Blanpied et al., 1995; Svantesson et al., 1994). These velocities were chosen because they closely match the movement velocities of the knee and ankle joints during physical activity (Blanpied et al., 1995). A greater enhancement of concentric muscle torque after eccentric contraction is achieved at higher movement velocities due to an enhanced influence of muscle pre-activation (Svantesson and Grimby, 1995). Increased movement velocity could also increase the stiffness of elastic contractile components in series during the eccentric phase (Nicol et al., 2006; Turner and Jeffreys, 2010). Thus, in eccentric loaded contractions, this increased musculotendinous stiffness in response to a high velocity can lead to increased elastic energy and its consequent release during the eventual muscle concentric phase. Notably, tendon stiffness could be tendon dependent. For example, the stiffness of the quadriceps tendon is lower than the Achilles tendon (Mabe and Hunter, 2014). Since the stiffness of the tendon is vital for storing and the later releasing of the eccentric-contraction associated energy (Ishikawa and Komi, 2004), joint-specific differences in tendon stiffness could affect the enhanced concentric muscle torque differentially.

Thus, the purpose of our study was to compare the knee extensors versus ankle plantar flexors differences of the enhanced concentric muscle torque after maximal eccentric contraction at two different movement velocities (120°/s and 180°/s) in healthy young adults. We hypothesized that the enhanced concentric muscle torque would be greater for the ankle plantar flexors versus knee extensors, and that it would be greater at 180°/s compared to 120°/s for both the joints.

## Methods

### Participants

A power analysis using ANOVA repeated measures, within factors model with effect size (*f*) of 0.3, Type I error (α) at 0.05, power (1-β) of 0.8, yielded 20 as the total number of required participants for our current study. Twenty-two healthy young adults (10 females; 12 males; age = 25.75 ± 3.03 years; body mass = 71.90 ± 16.34 kg; body height = 1.70 ± 0.91 m) voluntarily participated in this study. Our participants were typical college-going young adults. None of our participants were athletes or undergoing athletic training for any competitive or non-competitive sport participation. The Institutional Review Board at the University of Alabama at Birmingham, USA, approved this cross-sectional study and all participants provided informed consent before participating in this study. Eligible participants included healthy adults aged between 21 and 35 years. Participants were excluded if they had a history of a fracture in the past six months, metals/pin/screws inside their body, medical diagnoses involving the heart, skeletal muscle, bone, or had any other medical conditions which could limit their independent ambulation and/or full participation in our study.

### Measures

Physical characteristics such as body height and mass of participants were measured to the nearest 0.01 m and 0.1 kg on a stadiometer (Seca 217, Hamburg, Germany) and weighing scale (Garmin Index Smart Scale, Garmin, Olathe, KS), respectively. Participants wore light clothes with no shoes and emptied pockets during body height and body mass assessments.

We used a customized 3-question leg dominance questionnaire (which leg would you use to kick a ball (a), which leg would you use to squash a bug (b), and which leg would you use to take the first step (c)) to determine the leg dominance of participants.

We tested the maximum concentric strength of the non-dominant knee extensor and ankle plantar flexor muscles with and without maximal eccentric contraction, as described below. A total of three successful trials for each movement velocity and for each joint were collected, and then we chose the maximum muscle torque value for respective movement velocity for the analysis.

### Design and Procedures

Our participants performed a warm-up that included 5 min of cycling on a bicycle ergometer (Monark Exercise, Vansbro, Sweden) at a comfortable pace (25–50 watts) before any testing. We used an isokinetic dynamometer (System 3, Biodex Medical Systems Inc., Shirley, NY) for all tests. Tests were randomized to avoid any order effect. For all testing, we used standardized language during instruction and motivation delivery. We provided feedback on maintaining good posture during the strength tests. A familiarization session was performed, and a successful demonstration of the muscle strength tests was performed by the participant before the testing session.

### Knee Extensors Tests

We tested the knee extensors muscle maximal concentric strength with and without maximal eccentric contraction. Participants sat straight (85° from the horizontal) with the dynamometer shin pad attached to the lever arm, strapped on the tested leg 10–12 cm proximal to the ankle. Seat straps stabilized the contralateral leg and the trunk. Participants held handlebars attached to the seat when testing. The rotation axis of the dynamometer was aligned with the knee joint line. We secured the participant’s knee to the dynamometer knee attachment at 90° of knee flexion (where 0° is full knee extension). After securing the knee, we instructed our participants to “push” as hard and as fast as possible against a moving plate from 90° of knee flexion to 40° of knee flexion to elicit the only concentric condition (CONC). Thus, we assessed muscle strength for a total of 50° of range of motion of the knee in our study. We avoided muscle strength testing over a greater range of motion to minimize any risk of knee injury as the single-maximum eccentric contraction is associated with the risk of muscle damage (Margaritelis et al., 2021). With the same experimental set-up, for the maximal eccentric preloaded concentric condition (EccCONC), we secured the participant’s knee to the knee attachment at 40-degree of knee flexion. We then asked our participants to “push” as hard as possible against a plate that moved towards 90° of knee flexion. At 90° of knee flexion, the plate reversed its moving direction and moved toward extension, in line with the participant’s pushing force, while the participant was instructed to “keep pushing” as hard and as fast as possible. All these muscle strength tests were performed randomly at 120°/s and 180°/s.

### Ankle Plantar Flexors Tests

Participants sat straight (85° from the horizontal) with the tested ankle strapped and secured to the lever arm. Seat straps stabilized the trunk. The tested leg, with the knee fully extended, was stabilized at the thigh level with straps to minimize any contribution of the knee extensors during this test. Participants held handlebars attached to the seat during testing. The rotation axis of the dynamometer was aligned with the lateral malleolus of the participant’s tested leg. We strapped the participant’s ankle to the ankle attachment at 0° of dorsiflexion (neutral position of the ankle). We used a heel block to secure the heel and thus, prevent heel lift. After securing the ankle, we instructed our participants to push out as hard and as fast as possible against a moving plate from 0° of dorsiflexion to 25° of plantarflexion for the CONC. With the same set-up, for the EccCONC, we secured the participant’s ankle to the ankle attachment at 25° of plantarflexion. Then, we instructed our participants to ‘push’ as hard as possible against a moving plate while the plate moved to 0° of dorsiflexion. At 0° of dorsiflexion, the plate reversed its moving direction and moved toward extension (plantarflexion), in line with the participant’s pushing force, while the participant was instructed to “push” as hard and as fast as possible. All muscle strength tests were done randomly at 120°/s and 180°/s.

We provided our participants with a 60-s rest interval between each repetition. A standard verbal instruction of ‘push as hard and as fast as possible and maintain it until you hear stop’ was used with each participant before each strength test. A consistent level of motivation by the same instructor who tested the participants was delivered in the form of the instruction of ‘push harder’ during the performance of the tests. We selected a total of 50⁰ and 25⁰ of range of motion for muscle strength testing for the knee and the ankle to ensure the safety of our participants with the maximum eccentric preloaded concentric strength tests. Furthermore, these ranges are relative to each other as these ranges are approximately 50% of the range of motion of knee extension and ankle plantarflexion required for activities of daily living, respectively. All the data were exported from the Biodex computer into another laboratory computer (Dell Tech., Round Rock, TX) with the analysis software.

### Statistical Analysis

Statistical Package for Social Sciences SPSS 25.0 software (SPSS Inc., Chicago, IL) was used to perform data analysis. Data normality was checked by performing kurtosis, skewness, and Shapiro-Wilk tests. Descriptive statistics are shown as the mean ± SD in Table 1. Sex-based descriptive statistics were compared using independent *t*-tests or Mann-Whitney tests based on the normal or non-normal nature of the variable, respectively. To be able to compare individuals of different body mass, peak torques were normalized to body mass for each condition using customized codes in Microsoft Excel (Microsoft, Redmond, WA). We used separate two-way (joints x velocity) with repeated measures ANOVA to calculate joint-specific differences (knee vs. ankle) of (a) peak muscle torque of CONC, (b) EccCONC, and (c) concentric muscle torque enhancement (EccCONC/CONC) at both velocities of 120°/s and 180°/s. Significance for all the analyses was set at *p* < 0.05.

**Table 1:** Physical characteristics.

	Total participants (n = 22)	Men (n = 12)	Women (n = 10)
Age (years)	25.75 ± 3.05	25.98 ± 3.48	25.49 ± 2.60
Height (m)	1.71 ± 0.9	1.74 ± 0.08	1.67 ± 0.09
Body mass (kg)	71.9 ± 16.34	73.35 ± 14.19	70.15 ± 19.26
BMI (kg/m^2^)	24.64 ± 6.51	24.88 ± 4.15	24.34 ± 6.84
Sex (F/M)	10/12	12	10
Ethnicity (African American/ Caucasian/Asian/Latino/Middle Eastern)	4/8/1/1/8	2/2/0/0/8	2/6/1/1/0

Values are represented as means ± SD; Body mass index (BMI); F, female; M, male

## Results

### Participant Characteristics

The average age of our participants was 25.75 years with 10 females and 12 males. The average body mass index of our participants was 24.64 kg/m^2^. Ethnically, our population comprised of 8 Caucasians, 8 Middle-Easterns, 4 African Americans, 1 Asian, and 1 Latino, as shown in Table 1. There were no differences between men and women for age (*p* = 0.621), body mass (*p* = 0.659), and the BMI (*p* = 0.998); however, men tended to be taller than women (*p* = 0.051).

### Concentric Muscle Peak Torque with and Without Eccentric Preloading

**Figure 1:**
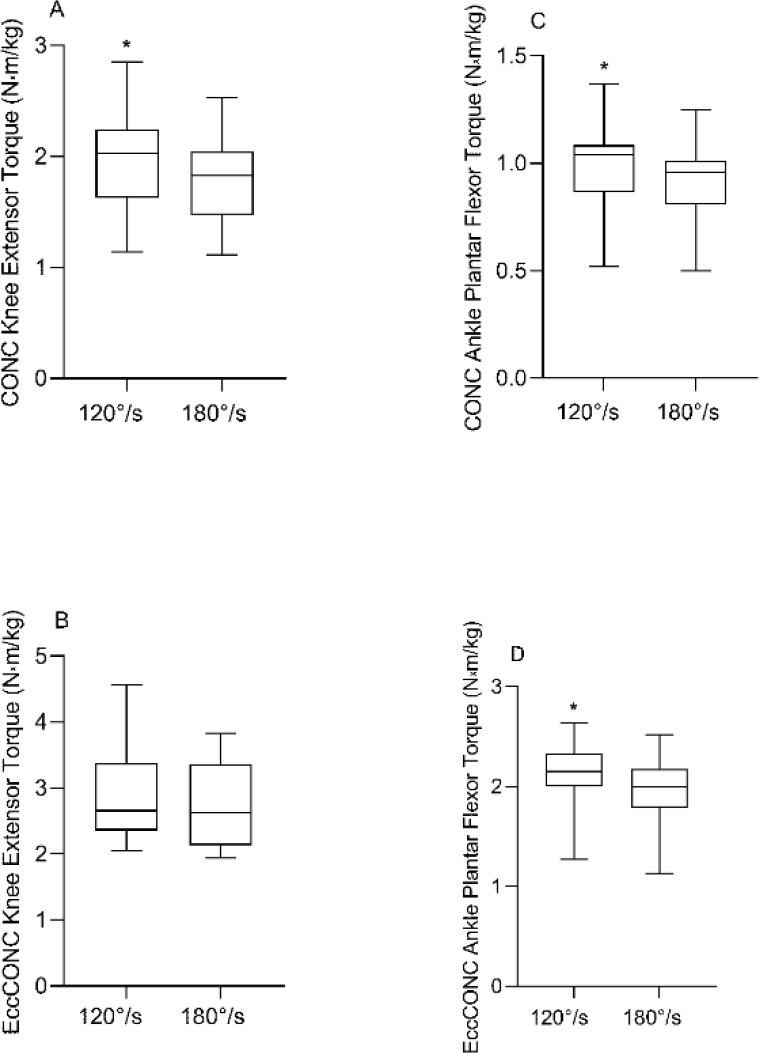
Differences in concentric (CONC) peak torque and eccentric preloaded concentric peak torque (EccCONC) between the velocities of 120°/s and 180°/s, for the knee extensors (A and B) and ankle plantar flexors (C and D), respectively. * Significance at p < 0.05

**Figure 2:**
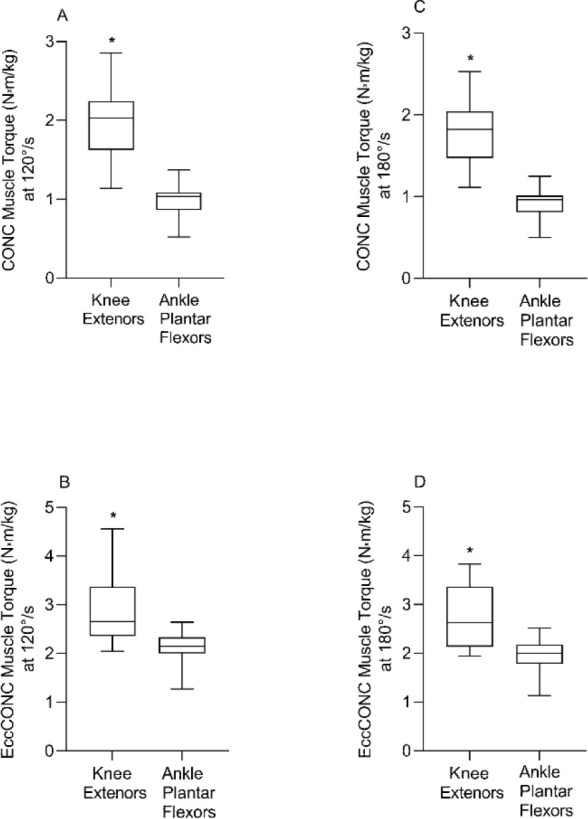
Comparison of concentric (CONC) peak torque and eccentric preloaded concentric peak torque (EccCONC) between the knee extensors and ankle plantar flexors, at 120°/s (A and B) and 180°/s (C and D), respectively. * Significance at p < 0.05

Differences in concentric peak torque with and without eccentric preloading for the knee extensors and ankle plantar flexors for both velocities of 120°/s and 180°/s are shown in Figures 1 and 2, respectively. The peak torque values of CONC were greater at 120°/s than 180°/s for both the knee extensors and the ankle plantar flexors (10.73% and 7.78%; *p* < 0.001 for both), respectively, as shown in Figure 1. Although the peak muscle torque values of EccCONC were greater at 120°/s than 180°/s for both the knee extensors and the ankle plantar flexors (4.04% and 8.12%), respectively; it was significant only for the ankle (*p* < 0.001) as shown in Figure 1.

We found joint-specific differences in the peak torque values of CONC with greater values for the knee extensors compared to ankle plantar flexors at both the velocity of 120°/s and 180°/s (102.00% and 96.67%; *p* < 0.001 for both), respectively (Figure 2). Similarly, peak torque values of EccCONC were greater for the knee extensors compared to the ankle plantar flexors at both velocities of 120°/s and 180°/s (32.86% and 38.07%; *p* < 0.001 for both), respectively (Figure 2).

### Concentric Muscle Torque Enhancement and Joint-Specificity

**Figure 3:**
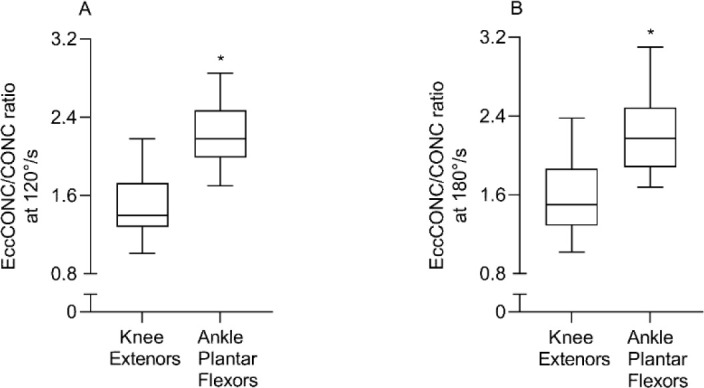
Differences in concentric muscle torque enhancement (EccCONC/CONC) between the knee extensors and ankle plantar flexors at 120°/s (A) and 180°/s (B); EccCONC, eccentric preloaded concentric peak torque; CONC, concentric peak torque. * Significance at p < 0.05

**Figure 4:**
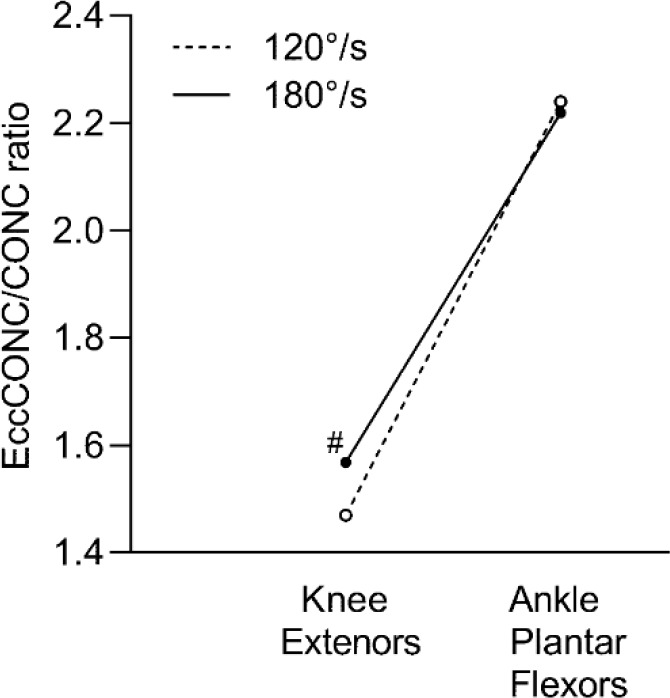
Differences in muscle torque enhancement (EccCONC/CONC) between 120°/s and 180°/s for the knee extensors and ankle plantar flexors. EccCONC, eccentric preloaded concentric peak torque; CONC, concentric peak torque # Significance trend at p = 0.07

Concentric muscle torque enhancement was significantly greater at the ankle plantar flexors versus the knee extensors at both movement velocities of 120°/s (52.35%; *p* < 0.001) and 180°/s (41.52%; *p* < 0.001) as displayed in Figure 3. Figure 4 shows the presence of a trend toward greater concentric muscle torque enhancement at 180°/s than 120°/s for the knee extensors (6.6%; *p* = 0.07) only.

## Discussion

To the best of our knowledge, this is the first study to compare concentric muscle torque enhancement after maximal eccentric loading for the knee versus ankle joints, at different velocities. The main finding of this study is that a greater concentric muscle torque enhancement was noted for the ankle plantar flexors compared to knee extensors. Our findings were not affected by sex since separate sex-based ANOVAs comparing CONC, EccCONC, and ECCCONC/CONC ratios revealed unchanged results from the overall result. Notably, the greater muscle torque of the knee extensors compared to the ankle plantar flexors did not translate into a greater enhancement of concentric muscle torque enhancement for the knee extensors. The greater torque enhancement of concentric muscle torque at the ankle plantar flexors, which had lower muscle torque compared to the knee extensors, may rely more on the contribution of the Achilles tendon, which is the strongest and largest tendon in the body (O'Brien, 2005). Since the tendon may be the primary site for storing potential energy during the SSC (Lichtwark and Wilson, 2007), a longer and thicker tendon, such as the Achilles tendon, can store greater amounts of elastic energy during the eccentric contraction resulting in greater concentric muscle torque enhancement. This is supported by previous data showing that tendon length is positively related to the concentric muscle torque enhancement (Hunter et al., 2015). Animal data suggest that distal muscles with short fibers and long tendons such as gastrocnemius can store more elastic energy than proximal muscles with longer fibers and shorter tendons such as quadriceps muscles (Alexander, 1984). Thus, it is possible that a longer Achilles tendon could generate greater concentric muscle torque enhancement than a shorter quadriceps tendon.

The timing of maximum eccentric torque development of the muscle during its eccentric phase could also, in part, explain our finding of greater concentric muscle torque enhancement at the ankle joint. Indeed, a previous finding by McCarthy et al. (2012) showed that greater muscle potentiation is significantly related to greater torque at the end of the eccentric phase of the SSC. Notably, in our study, the eccentric peak torque of the knee extensors versus ankle plantar flexors was reached earlier during the stretching phase of the SSC. Eccentric peak torque of the knee extensors reached 171.3 ms and 163.0 ms before the end of its eccentric phase at 120°/s and 180°/s, respectively, whereas the eccentric peak torque for the ankle plantar flexors occurred at 12.1 and 14.6 ms before the end of its eccentric phase at 120°/s and 180°/s, respectively. Accordingly, part of the elastic energy at the knee extensors could be lost before the shortening phase of the SSC began, which may contribute to the larger concentric muscle torque enhancement for the ankle plantar flexors compared to the knee extensors.

The concentric muscle torque enhancement at the knee extensors tended to be greater at the increased movement velocity of 180°/s in comparison to 120°/s (Figure 1). This is consistent with previous studies showing that enhancement of the SSC is greater at faster stretching velocity (Fukutani et al., 2015; Helgeson and Gajdosik, 1993; Svantesson and Grimby, 1995). Elastic contractile components become stiffer during high velocities of the stretch phase (Nicol et al., 2006; Turner and Jeffreys, 2010). This increased stiffness contributes to the storage of greater energy and consequent release of energy during the muscle shortening phase. We did not notice any movement velocity-related difference in concentric muscle torque enhancement at the ankle plantar flexors. This could have occurred because of a smaller absolute range of motion. A smaller range of motion at the ankle joint could potentially limit the muscle fiber recruitment required to reach a greater torque at a greater velocity of the eccentric phase. Lastly, the muscle fiber type may have an interaction with stretching velocity (Hunter et al., 2015). The ankle plantar flexors have a lower percentage of fast-twitch muscle fiber (type II) than the knee extensors (Gollnick et al., 1974). Since fast-twitch muscle fibers are critical for generating muscle torque enhancement (Hunter et al., 2015), a lower percentage of those fibers for the ankle plantar flexors could, in part, explain no difference in concentric muscle torque enhancement based on movement velocity.

Finally, our joint-specific finding related to concentric muscle torque enhancement is supported by previous studies (Fukunaga et al., 2001; Ishikawa and Komi, 2004), which concluded that different muscles behave differently with different velocities of the SSC (Nicol et al., 2006). Although the absolute concentric torque of the knee extensors was greater than the ankle plantar flexors in our study, greater concentric muscle torque enhancement of the ankle plantar flexors at both movement velocities (120°/s and 180°/s) might explain the uniqueness of the ankle joint in utilizing the SSC better than the knee joint. Thus, irrespective of the exact physiological mechanism responsible for concentric muscle torque enhancement, its difference being maintained throughout different stretching velocities at the ankle vs. the knee further supports our hypothesis of joint specificity of concentric muscle torque enhancement.

Our study has some limitations. First, although concentric muscle torque enhancement of the ankle plantar flexors was significantly greater than knee extensors, we do not know if this would apply at shorter range of motions or greater velocities since we only tested over a fixed range of motion and at two specific velocities (120°/s and 180°/s) for all the tests. Our study design relied on voluntarily generating maximum torque rather than a ‘pure’ physiological maximum torque. Thus, factors affecting the voluntary generation of maximum torque could limit the generalizability of our results. Testing a muscle over the ascending or the descending limb of the force-length curve could affect the output of muscle torque (Magid and Law, 1985); however, the operating range of optimal fiber length of muscles with long tendons such as gastrocnemius is markedly different from muscles with shorter tendons such as the vastus lateralis for a functional activity such as walking (Arnold and Delp, 2011). Thus, testing over the operating range of muscles at different joints versus testing over different limbs of the force-length curve could yield clinically useful information. Future studies should investigate if the joint specificity of concentric muscle torque enhancement is maintained separately over the operating range of different muscles. The type of muscle fiber concentration is one of the determinants of muscle torque enhancement (Hunter et al., 2015); however, this was not measured in the current study. Although we used similar movement velocities as in previous studies to assess concentric muscle torque enhancement, our results cannot be generalized to other greater movement velocities. Thus, future studies should examine if the results from our current study translate to other movement velocities.

Greater torque enhancement at the ankle than the knee could also occur due to other mechanisms which are not dependent on maximal eccentric torque. For example, a short-latency response of the muscle spindles of the ankle plantar flexors than the knee extensors (Cronin et al., 2011) could optimally regulate joint stiffness and yielding at the ankle, potentially helping with, greater torque enhancement at the ankle than the knee. However, we did not measure the short-latency response as that was out of the scope of our study. Finally, the limited sample size and the cross-sectional design nature of this study restrict the generalizability of our findings.

In conclusion, our study shows that the enhancement of concentric muscle torque preloaded with maximum eccentric torque is joint-specific in young adults. This is true for both men and women. Whether the joint specificity of concentric muscle torque enhancement after maximal eccentric loading differentially affects physical activity and sports performance is unknown. Alternatively, our study provides rationale for studying the relationship of joint-specific recovery after sports injuries with joint-specific muscle torque enhancement. Clinically, it would be beneficial to study the association between joint-specific enhancement of muscle torque and physical/functional activities such as walking, running, or balance in future research. Overall, our data provide a reference framework to investigate joint-specific concentric muscle torque enhancement for general and clinical athletic populations.
